# Microbial communities and their roles in the Cenozoic sulfurous oil reservoirs in the Southwestern Qaidam Basin, Western China

**DOI:** 10.1038/s41598-023-33978-3

**Published:** 2023-05-17

**Authors:** Yue Jiao, Liyun An, Wei Wang, Jian Ma, Chaodong Wu, Xiaolei Wu

**Affiliations:** 1 The Laboratory of Orogenic Belts and Crustal Evolution, School of Earth and Space Science, Peking University, Beijing, 100871 China; 2 College of Architecture and Environment, Sichuan University, Chengdu, 610065 China; 3 The No. 1 Oil Extraction Plant, Qinghai Oilfield Company, PetroChina, Haixi, 817000 Qinghai China; 4 College of Engineering, Peking University, Beijing, 100871 China

**Keywords:** Atmospheric chemistry, Geology

## Abstract

The latest discovery of sulfurous natural gas marked a breakthrough in the Cenozoic natural gas exploration in the southwestern margin of Qaidam Basin. The 16S rRNA analyses were performed on the crude oil samples from H_2_S-rich reservoirs in the Yuejin, Shizigou and Huatugou profiles, to understand the sulfurous gas origin, which was also integrated with carbon and hydrogen isotopes of alkane and sulfur isotopes of H_2_S collected from the Yingxiongling Area. Results show that the microorganisms in samples can survive in the hypersaline reservoirs, and can be classified into multiple phyla, including *Proteobacteria*, *Planctomycetes*, *Firmicutes*, *Bacteroidetes*, and *Haloanaerobiaeota*. Methanogens are abundant in all of the three profiles, while sulfate-reducing bacteria are abundant in Yuejin and Huatugou profiles, contributing to the methane and H_2_S components in the natural gas. The carbon, hydrogen and sulfur isotopes of sulfurous natural gas in the Yingxiongling Area show that the natural gas is a mixture of coal-type gas and oil-type gas, which was primarily derived from thermal degradation, and natural gas from the Yuejin and Huatugou profiles also originated from biodegradation. The isotopic analysis agrees well with the 16S rRNA results, i.e., H_2_S-rich natural gas from the Cenozoic reservoirs in the southwest margin of the Qaidam Basin was primarily of thermal genesis, with microbial genesis of secondary importance.

## Introduction

Hydrogen sulfide (H_2_S) is one of the non-hydrocarbon gases commonly found in carbonate reservoirs. The acidity of abundant H_2_S will cause corrosion and sulfide deposits in the pipelines during gas extractions^1,2^, and the toxic H_2_S may endanger the successful completion of the operation^3,4^. Therefore it is of great challenge to develop deeply-buried carbonate reservoir under complex geological conditions, e.g., high temperature and pressure, high H_2_S and CO_2_ content^5,6^, and it is essential to assess the concentration of H_2_S before drilling^7^. The H_2_S-rich gas resource in China is up to 1 × 10^10^ m^3^, which is widely distributed in large sedimentary basins with thick carbonate reservoirs, e.g., Bohai Bay Basin, Sichuan Basin, Tarim Basin and Ordos Basin^8,9^. The current researches on sulfurous natural gas mainly focus on compositions and isotopes of sulfur compounds and H_2_S origin^6,10–12^. Three primary H_2_S sources can be identified based on the burial depth and thermal maturity^13^: bacterial sulfate reduction (BSR), thermochemical sulfate reduction (TSR) and thermal decomposition reaction (TDR)^14,15^. Also, a small amount of H_2_S are derived from tectonic activities and volcanic eruptions, where H_2_S is not stable during migration and preservation^12,16^. The BSR is one of the main contributors to abundant H_2_S in oil and gas reservoirs^17^, with organic matter and sulfate as reactants^10,18^. BSR generally occurs in shallow-buried reservoirs with the participation of sulfate-reducing bacteria under low temperature (< 60 °C, with the peak at 20–40 °C), its effect on the gas generation in reservoirs is a research hotspot in recent years ^19,20^.

The Qaidam Basin is a huge petroliferous basin with an average elevation of ~ 3000 m (9800 ft) inside the Tibetan Plateau, northwest China^21,22^. It can be structurally divided into the northern fault block, the western depression and the eastern depression. Huge natural gas resource potential has been discovered in the Qaidam Basin^23,24^, including the Jurassic coal-type gas at the northern margin, the Paleogene-Neogene oil-type gas in the southwest, and the Triassic biogenetic gas in the Sanhu area^25–28^. The natural gas in the southwest is mainly originated from four sources: oil-associated gas from sapropel-type kerogen (type I kerogen), mixed oil-associated gas, coal-type gas and mixed gas^5,29^. Recently, the S58 well and SX58 well, high-sulfur gas producers, were discovered in the Yingzhong Area in the southwestern margin of the Qaidam Basin, with H_2_S volume fractions of 1.74% and 2.75%, respectively, while reservoirs in the western Qaidam Basin were low in sulfur content^30^. The western Qaidam Basin underwent an epeiric sea–continent transition from Paleozoic to Mesozoic^31,32^, and was an anoxic and hypersaline semi-deep lake in the Paleogene, where developed a small-scale invasion with widely-distributed salinization deposits^33–36^. Gypsum and source rocks are interbedded in the Lower Ganchaigou Formation in the Yingxiongling Area^37,38^, where sulfur isotopes are heavier than those of contemporaneous seawater due to hydrocarbon or sulfate reduction of microorganisms^5,12^.

Oil reservoirs are commonly characterized by high temperature, high salinity, and high pressure, where microorganisms (including bacteria and archaea) are prevailing, e.g., denitrifiers, sulfate-reducing bacteria, methanogens^39–42^. They take hydrocarbon, sulfur- and nitrogen-containing compounds in crude oil and deposits as energy sources with different survival strategies^43,44^, e.g., using specific membrane or cell walls to avoid the migration of high-concentration salt, pumping out ions and adjusting the osmotic pressure of interior/external cells^45,46^. Currently, the 16S rRNA sequencing technology allows for species level determination and improves the efficiency and accuracy of microbial community identification^47^, and has been applied to analyze function and distribution of sulfate-reducing bacteria, hydrocarbon degradation bacteria, methanogens, ferment bacteria, etc.^48–52^. In this study, the 16S rRNA sequencing and isotopic analysis were carried out on crude oil samples collected from Yuejin, Shizigou and Huatugou profiles in the southwestern margin of the Qaidam Basin, to (1) describe the metabolism pathways of microbial communities, (2) decipher the gas components and carbon, hydrogen, sulfur isotopic compositions of natural gas in the Yingxiongling Area, and (3) discuss the origination of sulfurous natural gas in southwestern Qaidaim Basin.

## Geological setting

**Figure 1:**
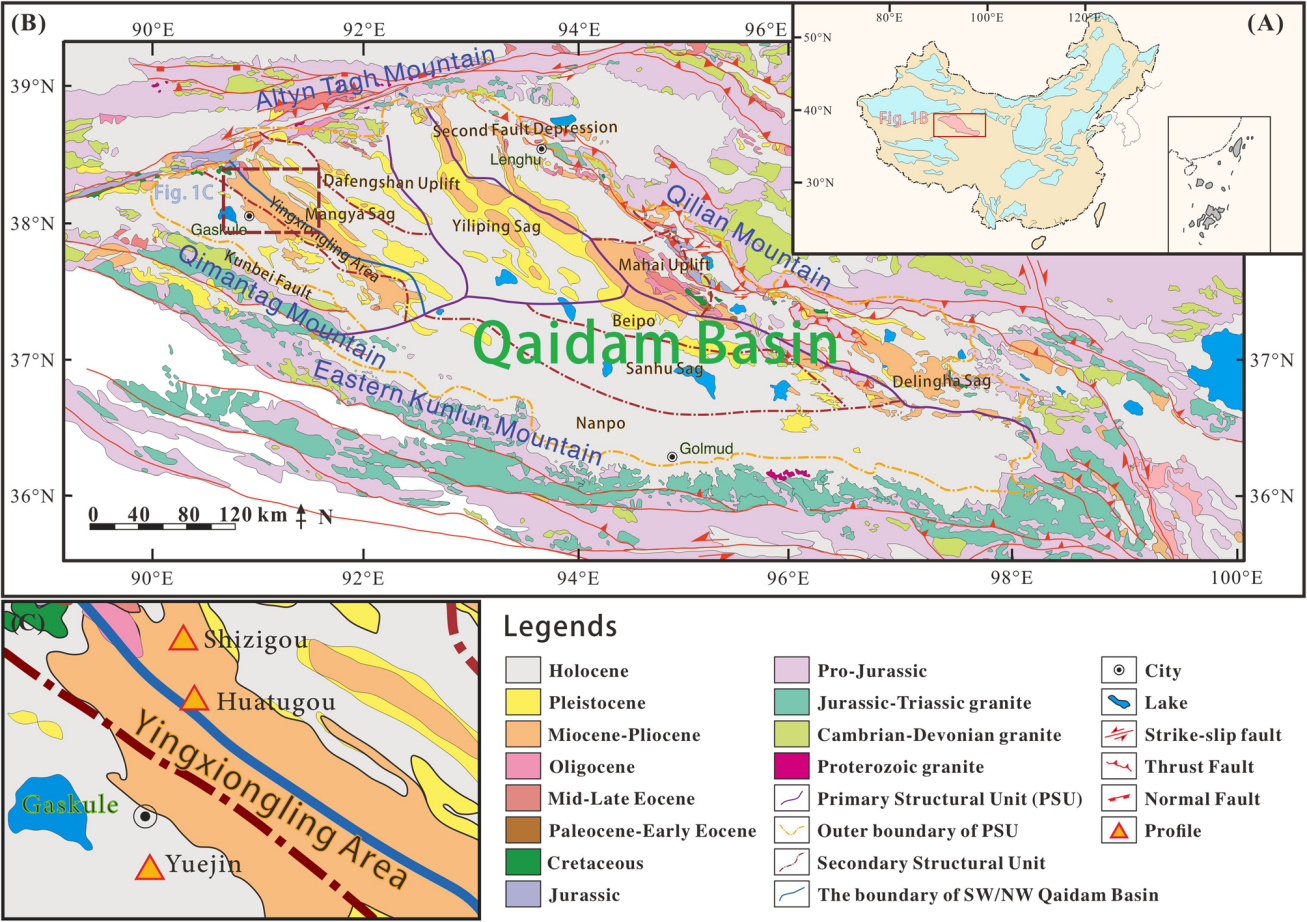
The geological map of the Qaidam Basin with sampling locations (**A**) The location of Qaidam Basin in China, modified after Zou et al.^22^, (**B**) Geological map of the Qaidam Basin, modified after Cheng et al. ^53^, (**C**) Sampling profiles in the southwestern Qaidam Basin.

The Qaidam Basin, a NWW-extending irregular triangle basin, is located in the north of the Qinghai-Tibet Plateau, which is bounded by the eastern margin of the Altun Mountains in the west and the southern margin of the Qilian Mountains in the east^53^, Fig. 1). It is a typical Mesozoic-Cenozoic continental petroliferous sedimentary basin, with area and an average altitude of 12.1 × 10^4^ km^2^ and 2900 m, respectively^54,55^. Large-scale strike-slip and rotation have occurred due to the activities of the Qinghai-Tibet Plateau since the Cenozoic^56^, where the subsidence center has been shifted from west to east, developing an unified large subsidence center in the Sanhu area in the east during the Quaternary^57,58^. Consequently, the Jurassic freshwater lacustrine source rocks were developed in the northern margin, the Paleogene-Neogene saline lacustrine source rocks were deposited in the western margin, and the Quaternary biogenic-gas source rocks were formed in the eastern margin^59^.

The Paleogene and Neogene source rocks in the western Qaidam Basin are dominated by lacustrine dark mudstone and calcareous mudstone with gypsum and salt beds^37,60^, the salinized deposits mainly originated from hot-arid climate with high evaporation^36^, which is mainly oil-prone, and generates low-mature to mature oil with minor gas production^61,62^. However, the proved natural gas reserves are currently far from the estimated geological natural gas resources, indicating potential gas exploration prospect^30,63^. The Yingxiongling Area is located in the west part of Mangya Depression, which is characterized by well-deformed folds and major faults^54^. The large-scale detachment faults connect the high-quality Paleogene source rocks with middle-shallow buried structural traps, while the middle-shallow faults possess good lateral plugging performance which are favorable for hydrocarbon preservation^64^. A large number of oil reservoirs were developed in the Yingxiongling Area, including the Shizigou, Huatugou, Youshashan and Yingdong reservoirs^65^. The structural pattern of the Yingxiongling Area varies significantly from west to east. It is characterized by a double-layer structure in vertical in the middle and the west, i.e., the upper layered salt rocks can work as a high-pressure cap rocks, and fractures and joints at the subsalt reservoirs are conducive to oil and gas migration, where CH_4_ is mainly derived from crude-oil-associated gas^62,66,67^. The eastern part of the Yingxiongling Area lacks salt caprocks (mainly halite) and possess a relatively simple structural pattern, with episodic charging of petroleum occurred from deep to shallow, in which natural gas is the by-product of condensate oil^56,68^. Recently, oil reservoirs containing high levels of H_2_S have been discovered in the middle of the Yingxiongling Area, this is different from the conventional reservoirs containing low sulfur content in former studies. Tian et al.^12^ suggested that the H_2_S in Yingxiongling Area is mainly derived by TSR, and the high temperature (approximately 181 °C) and burial depth (> 4500 m) are not favorable for BSR, but the origin of H_2_S still requires further discussion.

## Samples and methods

Crude oil samples were taken from sulfurous oil reservoirs from the interval of Eocene to Neogene, in the Yuejin, Shizigou, and Huatugou profiles. The sampling profiles are shown in Fig. 1C, and the sample information are listed in Table 1. Most of the samples are heavy oil, while the sample H-2 from Huatugou profile is light oil with high level of H_2_S. The 16S rRNA analysis were performed on these samples. The carbon, hydrogen and sulfur isotopic compositions of the sulfurous natural gas in Huatugou profile are tested.

**Table 1:** Information of sulfurous crude oil sampled from the southwest of the Qaidam Basin.

No	Profile	Well	Interval	Depth/m	Note
Y-1	Yuejin	Yueqian 1–71-21	the Lower N_1_	2575–2599	Heavy oil
Y-2	Yuejin	Yue 17–28	the E_3_^1^	3279–3475	Heavy oil
Y-3	Yuejin	Yueqian 1–49-1	the Lower N_1_	2450–2550	Heavy oil
S-1	Shizigou	Shixin 41H4-3–510	the E_3_^2^	4142–4839	Heavy oil
S-2	Shizigou	Shi41H-1–1-506	the E_3_^2^	4384–5133.5	Heavy oil
H-1	Huatugou	N13-6–5	the E_3_^2^	1479–1621	Heavy oil
H-2	Huatugou	SX58	the E_3_^2^	5502–5514	Light oil, high H_2_S

### 16S rRNA analysis

To extract total microbial genomic DNA of crude oil, we added three volumes of isooctane (2,2,4-trimethylpentane), mixed thoroughly, and let it stand overnight at room temperature. The precipitates were obtained after centrifuged at 5000 × *g* for 30 min at 4 °C, washed, and re-suspended twice with three volumes of isooctane, then centrifuged at 5000 × *g* for 30 min at 4 °C. After dried in vacuum oven at 55 °C, for 2 h, the precipitates were collected for the total microbial genomic DNA extraction by using FastDNA® SPIN Kits for Soil (Axygen Biosciences, USA; MP Biomedicals, USA) following the procedures in accordance with the manufacters' instructions. To be detailed, (i) Sodium Phosphate Buffer and MT Buffer were used to protect DNA during the cell-wall-breaking by lysozyme, (ii) Buffer G-B, DV, and BV were used to remove cell walls, lipids, and proteins, respectively; (iii) Buffer W1, W2 and Eluent were utilized to collect nucleic acid molecules.

### Sequencing and data analysis

The V3—V4 region of bacterial 16S rRNA genes were amplified using 338F (ACT CCT ACG GGA GGC AGC AG) and 806R (GGA CTA CHV GGG TWT CTA AT) primers. The PCR products were purified and quantified, and then were sequenced on Illumina MiSeq platform as described previously^69^. The acquired sequences were filtered for quality control as previously described^70^. To achieve what, all chimeric sequences were removed using the USEARCH tool based on the UCHIME algorithm 2,3. Sequences were then split into operational taxonomic units (OTUs) based on a threshold of 97% identity between nucleotide sequences using the UPARSE pipeline^71–73^. In addition, OTUs with fewer than two sequences were removed. Each bacterial OTU representative sequence was assigned taxonomy against the Silva database (release 128^74^. OTUs annotated to bacterial domain were retained, and OTU tables were resampled to a minimum number of sequences.

### Natural gas isotope measurement

The H_2_S-rich natural gas was collected from the Well SX58 in the Huatugou profile in the southwest of Qaidam Basin. The H_2_S in natural gas was solidified with cadmium acetate solution (25 g cadmium acetate solid was dissolved in 3.5 mol/L HAc per liter) to generate yellow cadmium sulfide precipitate based on the sulfur component separation method^75^. After that, black silver sulfide precipitates were produced by adding 0.05 mol/L silver nitrate solution and was washed with distilled water. The sulfur isotope analysis was completed at the Chinese Academy of Geological Sciences. The carbon and hydrogen isotope tests of natural gas were conducted at the China Petroleum Exploration and Development Research Institute. Experiment details can be referred from previous studies^76^.

## Results and discussion

### Abundance of different microbial communities

**Figure 2:**
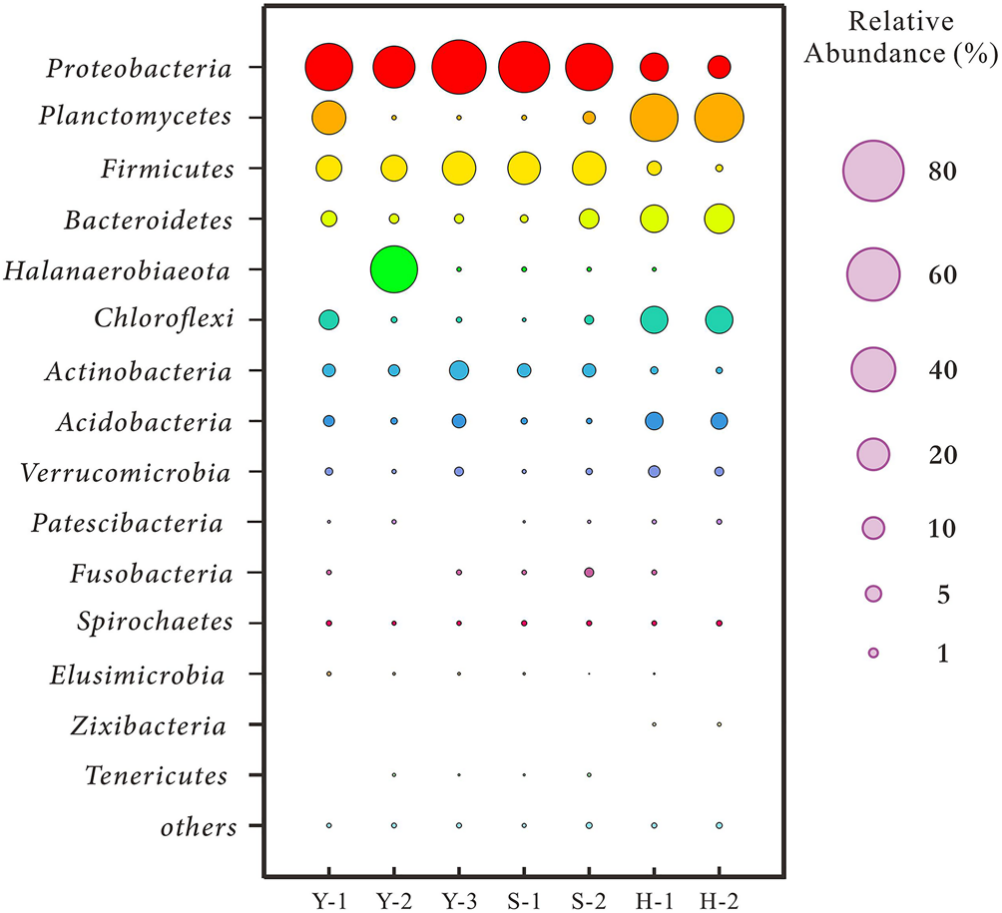
Bubble diagram of top 15 Phyla in samples from the Yuejin, Shizigou and Huatugou profiles.

**Figure 3:**
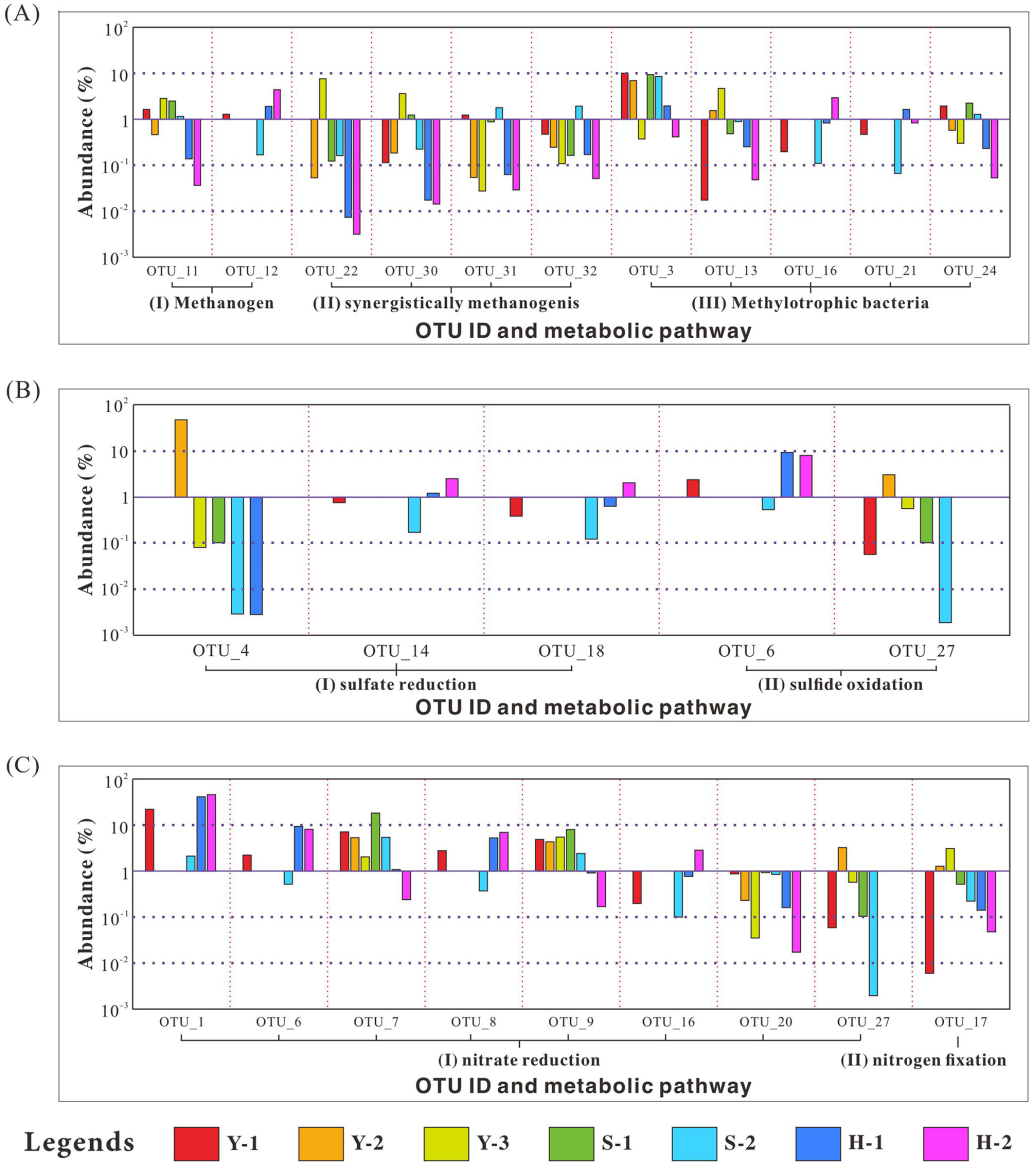
Histogram of microbial abundance involved in methane, sulfate and nitrate metabolism in the Yuejin, Shizigou and Huatugou profiles.

A total of 1338 OTUs were identified via the 16S rRNA sequencing from seven samples in the three profiles in southwestern Qaidam Basin. After abundance normalization, top 15 Phyla, including *Proteobacteria, Planctomycetes*, *Firmicutes*, *Bacteroides*, and *Haloanaerobes*, were identified with abundance variations shown in Table S1 and Fig. 2. The top 32 OTUs, with abundance accounting for about 80% of the total value, were selected for the next analysis. These bacteria typically thrive depending on hydrocarbons^77–80^, nitrogenous compound^81^, and sulfur compound^82^, and can survive in the hypersaline and alkaline environment^83–86^. Their specific abundance, scientific name and metabolic behaviors are listed in Table S2. Among them, methanogens, sulfate-reducing bacteria and nitrate-reducing bacteria, which are widely spread in global oil reservoirs^87–89^, were unevenly distributed in the crude oil samples from the southwestern Qaidam Basin (Fig. 3).

#### Methanogens

Among the top 32 OTUs, eleven of them are involved in methane metabolism, which can be divided into three categories: methanogen (I), synergistically methanogenesis (II) and methylotrophic bacteria (III). They are unevenly distributed in the Yuejin, Shizigou and Huatugou profiles (Fig. 3A). The type I is methanogen, including OTU_11 and OTU_12, which can be classified into *Actinobacteria* and *Chloroflexi*, respectively. They are frequently observed at high-temperature oil reservoirs^90,91^ and can degrade long-chain n-alkanes into methane^92,93^. The OTU_11 abundance is high in the Yuejin and Shizigou profiles (1.15–2.8%), while the OTU_12 abundance is high in the Huatugou profile (up to 4.29%), whose occurrence might contribute positively to methane generation at reservoirs in the southwestern Qaidam Basin. The type II, OTU_22 and OTU_30 of the genus *Bacillus*, can produce surfactants to promote the formation of methane hydrate and enhance methane migration^94^, with thermostability and salt-tolerance^95^. Surfactants derived from OTU_31 and OTU_32 of the genus *Pseudomonas* can emulsify aliphatic compounds and aromatic hydrocarbons and cooperate with primary producers to promote methanogenesis^96^. These bacteria abundance are high in samples Y-3, S-1, and S-2, indicating that the methane migration associated with microbial community also enhanced methane enrichment in Yuejin and Shizigou profiles. Different from that, OTU_3, OTU_13, OTU_16, OTU_21, and OTU_24 can use hydrocarbons, e.g., methane, and carbohydrate, as carbon sources for heterotrophy or autotrophy, consuming methane in the environment^97–101^. All these bacteria are observed from three profiles, but are unevenly distributed in space. The OTU_3, OTU_13 and OTU_24 are relatively rich in the Yuejin and Shizigou profiles, while OTU_16 and OTU_21 possess high abundance in the Huatugou profile, confirming sufficient methane supply in these three profiles with different microbial communities.

#### Sulfate-reducing bacteria

Five OTUs are involved in the metabolism of sulfur compounds, including sulfate reduction (I) and sulfide oxidation (II), respectively. They also unevenly distributed in the Yuejin, Shizigou, and Huatugou profiles (Fig. 3B). The type I includes OTU_4, OTU_14, and OTU_18, and can be classified into three phyla: *Halanaerobiaeota*, *Proteobacteria*, and *Bacteroidetes*. They can survive in saline-alkaline soil and hot spring with the temperature range of 4–92 °C and the pH range of 2.3–10.6^102,103^, taking organic matter as the electronic donor to reduce sulfate, sulfite or thiosulfate to sulfide^82,104^. The abundance of OTU_4 is extremely high in the sample Y-2 from the Yuejin profile (up to 47.37%), while OTU_14 and OTU_18 abundance are high in the sample H-2 from the Huatugou profile (2.57% and 2.12%, respectively). The OTU_14 can conduct anaerobic oxidation of methane (AOM) with methanogens, producing H_2_S and decreasing CH_4_ content^105^. It agrees well with the high H_2_S content of the sample H-2 and is responsible for the H_2_S occurrence at the sulfurous reservoirs in the Yuejin and Huatugou profiles. The type II bacteria, including OTU_6 and OTU_27, are sulfide-oxidizing nitrate-reducing bacteria (soNRB), can oxidize sulfide into sulfur or sulfate based on the proportion between nitrate and sulfide in reservoirs^106^. The OTU_6, belonging to the genus *Bacteroides*, can take H_2_S as the electron donor for denitrification reaction and oxidize H_2_S to sulfur and further generates sulfate^107^. The OTU_6 can be detected in three profiles, and is abundant in two samples from Huatugou profile, with relative abundance of 9.4% and 8.24%, respectively. The OTU_27, belonging to the genus *Comamonas*, can transform sulfide in the environment into sulfur^108^, with abundance of 3.28% in the Y-2 sample, but was not detected from samples in the Huatugou profile. Sulfate minerals (e.g., celestite, barite, gypsum, and mirabilite) and sulfides (e.g., framboid pyrite), which regionally enriched in the Paleogene in the Yingxiongling Area^36,109^, not only provided substrates for bacterial sulfate-reduction reaction, but also resulted in difference in sulfate-reducing bacteria abundance and sulfide-oxidizing bacteria abundance among the Yuejin, Shizigou, and Huatugou profiles.

#### Nitrate-reducing bacteria

Nine OTUs are involved in the metabolism of nitrogen-containing compounds, including nitrate reduction (I) and nitrogen fixation (II), respectively, whose abundance was different in the Yuejin, Shizigou, and Huatugou profiles (Fig. 3C). The type I is nitrate-reducing bacteria (NRB), including OTU_1, OTU_6, OTU_7, OTU_8, OTU_9, OTU_16, OTU_20 and OTU_27, which can trap electrons from organic carbon (e.g., methane, amino acid, carboxylic acid, aromatic hydrocarbon^110–112^, and reduce nitrate following an order: NO_2_^−^ → NO → N_2_O → N_2_ → NH_4_^+^^113,114^. These microorganisms are abundant in the Yuejin profile, and show different abundance in Huatugou and Shizigou profiles. Specifically, the abundance of OTU_1, OTU_6, OTU_8 and OTU_16, are high in the Huatugou profile, while the abundance of OTU_7, OTU_9, OTU_20 and OTU_27 are high in the Shizigou profile. Furthermore, although OTU_6 and OTU_16 belong to NRB, they can use H_2_S and CH_4_ as electron donors to generate sulfate and CO_2_, respectively^97,107^, the enrichment of these bacteria confirmed the sufficient supply of H_2_S and CH_4_ in the Huatugou profile. The OTU_17 belongs to the nitrogen-fixing bacteria (type II), and can transform nitrogen into nitrate^115^, it is ecologically balanced with various nitrate reducing bacteria, and unevenly distributed at these three profiles.

Summarily, microbial communities with different metabolic behaviors are separated from crude oil samples in the Yuejin, Shizigou and Huatugou profiles, while methanogens, sulfate-reducing bacteria and nitrate-reducing bacteria abundance varied greatly among different profiles. These microorganisms can survive in high-temperature and hypersaline reservoirs, react with hydrocarbon, sulfate and nitrate to generate methane, H_2_S and various nitrogenous compounds, respectively, indicating that microorganisms contributed greatly to the Cenozoic H_2_S-rich natural gas in the southwestern margin of the Qaidam Basin.

### Isotope distribution pattern of natural gas

The carbon, hydrogen and sulfur isotopic tests were conducted on the sulfurous natural gas from Well SX58 in Huatugou profile, and are compared with the collected data in the Yingxiongling Area from references. The hydrocarbon compositions of natural gas from the Huatugou profile is about 93.4%, which is dominated by methane with a proportion of 85.9% (Table S3). In general, natural gas reservoirs are commonly dominated by alkanes (e.g., methane, ethane and propane^116^, and the compound-specific carbon isotope and hydrogen isotope of natural gas can provide significant insight into the alkane sources^117,118^. The carbon isotopes of methane, ethane, propane, butane and n-pentane in the tested sample increased successively with carbon number, which are − 39.730‰, − 28.915‰, − 25.288‰, − 24.715‰, and − 24.652‰, respectively. The hydrogen isotopes of methane and ethane are − 189.586‰ and − 141.185‰, respectively. The non-hydrocarbon component in natural gas accounts for about 5.6% and is dominated by H_2_S (with a proportion of 2.8%, and sulfur isotope of 32.2‰). This is close to the H_2_S content (2.75%) and sulfur isotope value (32.5‰) in the middle Yingxiongling Area^5^.

**Figure 4:**
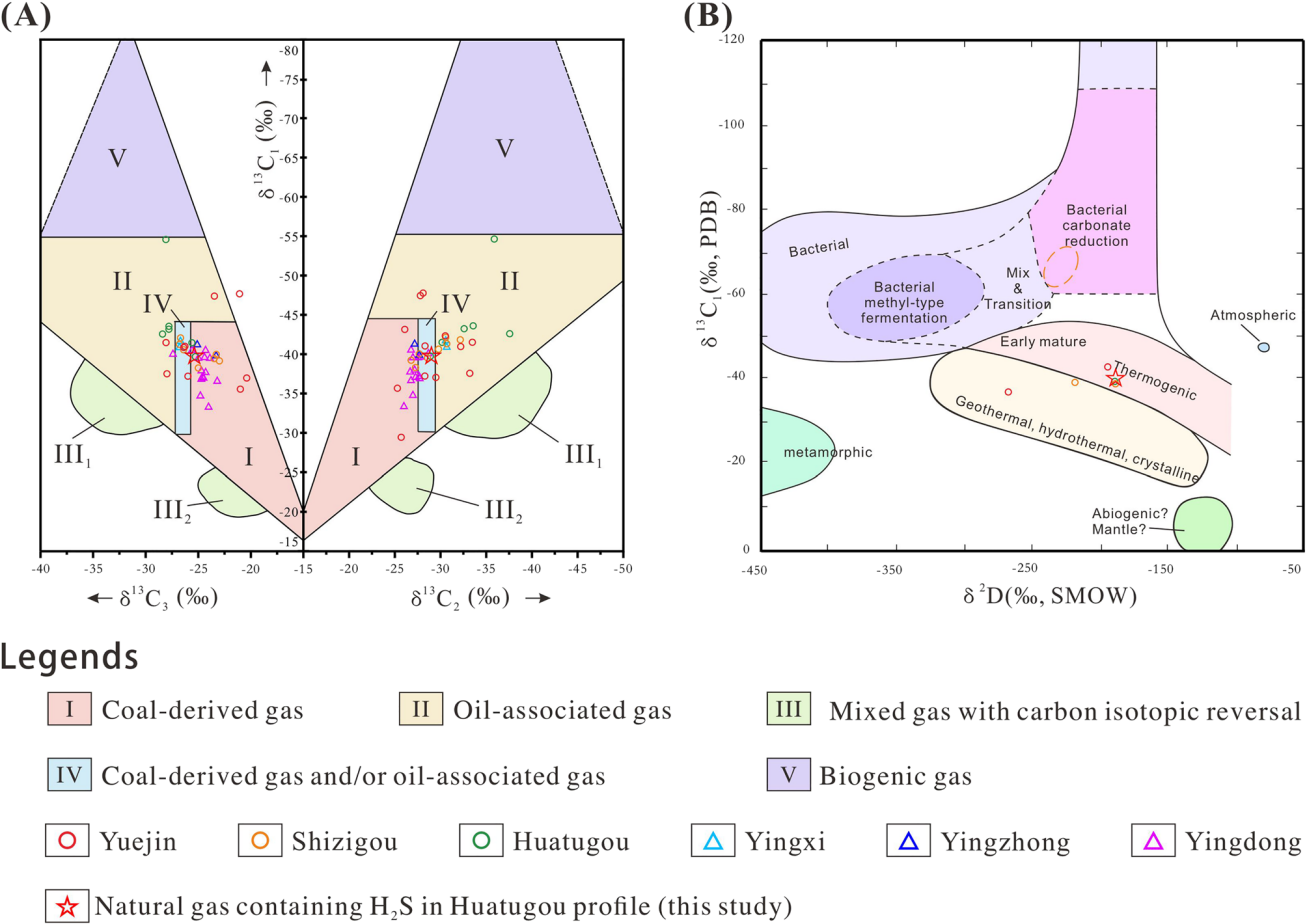
Carbon and hydrogen isotopes of natural gas in the Yingxiongling Area, southwestern Qaidam Basin. (**A**) carbon isotopes; (**B**) hydrogen isotopes, modified after Feng et al.^125^.

Generally, organic gas can be characterized by the increasing carbon isotope (δ^13^C) of alkanes with carbon numbers, while alkanes from inorganic gas, fractured carbonate rocks, tight reservoirs and coal gas present reversals in carbon isotopes^13,119^. Natural gas or carbonate generated by microbial reaction is generally low in ^13^C, because light isotope can be preferentially utilized by microorganisms^120^. The carbon isotopic values of methane, ethane and propane in the southwestern margin of the Qaidam Basin are in ranges of − 54.6‰ to − 29.4‰, − 37.6‰ to − 25.4‰, − 28.4‰ to − 13.1‰, respectively (Table S3^121–124^), which are defined as oil-type gas, coal-type gas and mixed gas, with no carbon isotopic reversals (Fig. 4A^125^).

Five samples (Table S3) with δ^13^C_1_ < − 45.5‰ were found in the Yuejin and Huatugou profiles by Chen et al.^76^, Liu et al.^126^ and Li et al.^127^. The lowest value was about -54.6‰ and was close to the value of biogenic gas (− 55‰) proposed by Ni et al.^128^. Biomarker analysis by Chen et al.^76^ confirmed that light hydrocarbons in some samples from the Yuejin and Huatugou profiles were derived from the degradation of crude oil by microorganism. It agrees well with the above analysis of microbial community in section "Abundance of different microbial communities". Furthermore, the hydrogen isotopes of methane in natural gas can be used to identify the natural gas origin. Deuterium isotope is gradually enriched with increasing maturity of natural gas, where δD of organic gas can be characterized by a positive sequence trend, which is similar to the carbon isotope distribution. Hence, the crossplot of δD and δ^13^C can distinguish the origin of methane^129,132^. Among the collected isotope data, few samples were performed with carbon and hydrogen isotopes at the same time, where the δD of methane was between − 267‰ and − 189‰. The measured δD of the sulfurous natural gas is − 189.586‰ (Fig. 4B). Combined with above carbon isotope values (− 54.6‰ to − 29.4‰), it can be confirmed that the natural gas was mainly derived from thermal degradation, at low-maturity stage to maturity stage. Compared with intensive negative ^13^C_1_ shifts in the Quaternary biogenic gas in the eastern Qaidam Basin (− 72.3 to − 60.53‰^126,130,131^, few samples from the Yingxiongling Area in the southwest of Qaidam Basin present minor negative drifts. Therefore, the H_2_S-rich natural gas in the southwest of Qaidam Basin was derived from both thermal degradation and biodegradation, with the former of the most importance.

Biogenic gas is generally derived from biological fermentation and biological reduction^132^. Compared with sulfate (the reactant), the preferential use of light isotopes by microorganisms can bring a negative shift of 15–25‰ to sulfur isotopes in the H_2_S of BSR origin^133,134^. The TSR requires sufficient sulfate supply and high temperature, where sulfur isotopes in the H_2_S do not drift greatly, usually between 5‰ and 15‰ ^8,16,135^. The isotopic data of H_2_S in the southwestern margin of the Qaidam Basin was poorly reported in previous studies. The tested sulfur isotope of H_2_S-rich natural gas in this paper is 32.2‰, which does not present significant fractionation compared with the sulfur isotopes (30.3–33.5‰) of equivalent sulfate minerals (e.g., anhydrite, glauberite^136^). Hence, the H_2_S from the sulfurous oil reservoirs in the southwest of Qaidam Basin is primarily of thermal genesis^12^, with microbial genesis of secondary importance.

The formation of H_2_S is an ongoing process from the geological period to the present^137^. The discussion on the H_2_S origin is beneficial for oil & gas operators in the Qaidam Basin, because it will not only improve the theory of hydrocarbon generation in sulfurous reservoirs, but also help to better discover the high-sulfur intervals in exploration and development.

## Conclusions

1. Microorganisms were extracted from crude oil samples from the Yuejin, Shizigou, and Huatugou profiles in the southwestern margin of the Qaidam Basin through integrating Axygen and MP FastDNA SPIN kits and were examined using 16S rRNA sequencing. Results show that the microorganisms vary greatly among these profiles, where methanogens and nitrate-reducing bacteria are well developed in the Yuejin, Shizigou, and Huatugou profiles, and sulfate-reducing bacteria are frequently observed in the Yuejin and Huatugou profiles. The spatial distribution of these microorganisms may be attributed to the different reservoir conditions, and contributed to the development of methane and H_2_S in the sulfurous reservoirs.
2. The isotopic pattern of hydrocarbon components show no obvious reversals in carbon isotopes, indicating that the Cenozoic natural gas in the Yingxiongling Area is a mixture of coal-type gas and oil-type gas. It is believed that the natural gas was mainly derived from thermal degradation with microbial genesis of secondary importance, which can be confirmed by hydrogen isotope distribution. No obvious fractionation occurred to sulfur isotopes of H_2_S when compared with equivalent sulfate minerals, suggesting that the BSR was not the primary origin for H_2_S in the sulfurous oil reservoirs.

## Supplementary Information

Supplementary Information.

## Data Availability

All data generated or analyzed during this study are included in this published article and its supplementary information files.
